# Hohe Krankheitslast bei Patienten mit ANCA-assoziierter Vaskulitis: Versorgungsdatenstudie in Deutschland

**DOI:** 10.1007/s00108-021-01181-z

**Published:** 2021-10-19

**Authors:** H. G. Haller, S. von Vietinghoff, P. Spearpoint, A. Deichmann, I. Buchholz, M. P. Schönermark, P. Rutherford, D. Götte

**Affiliations:** 1 Medizinische Hochschule Hannover, Hannover, Deutschland; 2 Vifor Fresenius Medical Care Renal Pharma, Flughofstraße 61, 8152 Glattbrugg, Schweiz; 3 SKC Beratungsgesellschaft mbH, Hannover, Deutschland

**Keywords:** Nierenerkrankungen, Lungenerkrankungen, Rezidiv/Vaskulitis, Morbidität/ANCA-assoziierte Vaskulitis, Mortalität/ANCA-assoziierte Vaskulitis, Kidney diseases, Lung diseases, Relapse/vasculitis, Morbidity/ANCA-associated vasculitis, Mortality/ANCA-associated vasculitis

## Abstract

**Hintergrund und Zielsetzung:**

Unter dem Begriff der mit antineutrophilen zytoplasmatischen Antikörpern (ANCA) assoziierten Vaskulitis (AAV) wird eine Gruppe seltener, chronischer, durch rezidivierende systemische Entzündungen gekennzeichneter Autoimmunerkrankungen mit vielfältigen Morbiditäten zusammengefasst. Patienten mit AAV leiden unter diversen Organmanifestationen und schweren Nebenwirkungen der Therapie. In dieser retrospektiven Studie wurde die konkrete Belastung der Patienten durch die AAV-Erkrankung in Deutschland untersucht.

**Methodik:**

Basierend auf anonymisierten Längsschnittdaten der gesetzlichen Krankenversicherung (GKV) zur medizinischen Versorgung zwischen 2013 und 2016 wurden aus einer repräsentativen Kohorte von etwa 3 Mio. Versicherten Patienten mit Granulomatose mit Polyangiitis (GPA) und mikroskopischer Polyangiitis (MPA) identifiziert und ausgewählte klinische Aspekte systematisch analysiert.

**Ergebnisse:**

Die häufigsten begleitenden Morbiditäten von GPA und MPA waren Nieren- und Atemwegserkrankungen. Eine schwere Nierenbeteiligung trat bei 11,6 % der GPA- und 24,3 % der MPA-Patienten innerhalb von 15 Quartalen nach der Diagnose auf. Bei einem Drittel der Patienten mit AAV entwickelten sich innerhalb der ersten 3 Quartale nach Diagnose schwere Infektionen. Die Rate der schweren Rezidive betrug jährlich 5–8 %. Patienten mit AAV und Nierenbeteiligung oder Infektionen zeigten zudem eine hohe jährliche Mortalitätsrate von 14,4 % bzw. 5,6 %.

**Schlussfolgerung:**

Anhand dieser Analyse deutscher Versorgungsdaten wurden krankheitsspezifische Annahmen der Belastung von Patienten mit AAV bestätigt und für den deutschen Kontext konkretisiert. Patienten mit AAV leiden unter einer hohen Morbiditätsbelastung, einschließlich multipler Krankheitsmanifestationen, Rezidiven und schwerer Komplikationen aufgrund der AAV-Therapie.

**Zusatzmaterial online:**

Die Online-Version dieses Beitrags (10.1007/s00108-021-01181-z) enthält eine zusätzliche Tabelle sowie zwei weitere Abbildungen.

## Abstract (en)

**Background & Objectives:**

Antineutrophil cytoplasmic antibody (ANCA)-associated vasculitis (AAV) represents a group of rare chronic autoimmune diseases characterized by recurrent systemic inflammation provoking multiple morbidities. AAV patients suffer from various organ manifestations and treatment-related severe adverse effects. This retrospective study investigated the concrete burden of AAV disease on patients in Germany.

**Methods:**

Based on anonymized longitudinal German statutory health insurance (SHI) claims data from the years 2011–2016, a representative cohort of approximately 3 million insured persons was used to identify patients with granulomatosis with polyangiitis (GPA) and microscopic polyangiitis (MPA), and selected clinical aspects were systematically assessed.

**Results:**

The most frequent concomitant morbidities of GPA and MPA were renal and respiratory disorders. Severe renal involvement occurred in 11.6% of GPA and 24.3% of MPA patients within 15 quarters of diagnosis. Severe infections developed in one third of AAV patients within the first three quarters post-diagnosis. The annual rate of major relapses was 5–8%. AAV patients with renal impairment or infections showed increased annual mortality rates of 14.4 and 5.6%, respectively.

**Conclusion:**

Based on this analysis of German health care data, disease-specific assumptions regarding the burden on AAV patients were confirmed and concretized for the German context. AAV patients suffer from a high burden of morbidity, including multiple disease manifestations, relapses, and severe complications due to AAV treatment.

Unter dem Begriff der mit antineutrophilen zytoplasmatischen Antikörpern (ANCA) assoziierten Vaskulitis (AAV) werden seltene, chronische und rezidivierende Autoimmunerkrankungen zusammengefasst, die eine potenziell lebensbedrohliche systemische Entzündung kleiner Blutgefäße verursachen. Die Patienten leiden an verschiedenen schweren Organmanifestationen wie Nieren- und Lungenbeteiligungen. Darüber hinaus kommt es unter der immunsuppressiven Behandlung, insbesondere mit hoch dosierten Glukokortikoiden, häufig zu Infektionen. Somit besteht für AAV-Patienten eine erhebliche Krankheitslast.

Die AAV umfasst eine Gruppe chronischer Autoimmunerkrankungen, die mit systemischen Entzündungen sowie Nekrosen in kleinen Blutgefäßen einhergehen [9, 11]. Eine AAV tritt episodisch rezidivierend auf und stellt unbehandelt eine schwere, potenziell lebensbedrohliche Erkrankung mit einer mittleren Überlebenszeit von nur 5 Monaten dar [24]. Die beiden häufigen Subtypen der AAV sind die Granulomatose mit Polyangiitis (GPA oder Wegener-Granulomatose) und die mikroskopische Polyangiitis (MPA), die in Bezug auf ihre klinischen, histologischen und serologischen Merkmale differenziert werden, jedoch aufgrund ihrer Gemeinsamkeiten mit meist identischen Therapieschemata behandelt werden. Die AAV sind seltene Erkrankungen mit einer geschätzten Prävalenz und jährlichen Inzidenz von 149 bzw. 12–16 Fällen pro 1 Mio. Menschen in Deutschland, bei etwa 70 % liegt eine GPA vor, bei etwa 22 % eine MPA [6, 16].

Eine AAV wird oft erst im fortgeschrittenen Stadium diagnostiziert, nachdem sich typische Symptome aufgrund von Organschädigungen entwickelt haben. Mögliche Manifestationen sind dabei [7]

- Nierenfunktionseinschränkungen,
- eine Beteiligung der Lungen (Lungenblutungen),
- Purpura,
- eine Beteiligung der Augen und des Hals-Nasen-Ohren-Trakts,
- neurologische Störungen sowie
- unspezifische systemische Symptome.

Eine AAV erfordert wiederholte Krankenhausaufenthalte, da durch den progressiven Charakter letztendlich ein Verlust der Organfunktion resultiert. Insbesondere kann eine Beteiligung der Nieren zu einer terminalen Niereninsuffizienz mit ungünstiger Prognose führen [2, 12].

Begleitende Morbiditäten wie auch deren Behandlung erhöhen die Krankheitslast

Zur Behandlung von AAV werden hauptsächlich Glukokortikoide in Kombination mit Cyclophosphamid oder Rituximab eingesetzt, um eine Remission zu erreichen [4, 5]. Allerdings kann die hoch dosierte wie auch die langfristige Einnahme von Glukokortikoiden aufgrund der Immunsuppression zu schweren Infektionen und anderen Nebenwirkungen führen [13]. Sowohl die diversen begleitenden Morbiditäten als auch deren Behandlung erhöhen die Krankheitslast und reduzieren die Lebensqualität der Patienten mit AAV erheblich [12, 22, 23].

Obwohl die wesentlichen Komplikationen der AAV bekannt sind, ist der derzeitige Wissensstand über deutsche Patienten mit AAV begrenzt [25]. Die vorliegende Versorgungsdatenstudie soll zu einem besseren Verständnis der medizinischen Belastung von Patienten mit AAV in Deutschland führen, da sie ausgewählte Aspekte, wie begleitende Morbiditäten, die Rate schwerer Rezidive und die Mortalitätsrate von Patienten mit GPA und MPA, systematisch untersucht.

## Methodik

### Datenquelle.

Es wurde eine nichtinterventionelle retrospektive Längsschnittdatenanalyse von Versorgungsdaten aus den Jahren 2013 bis 2016 durchgeführt, die von etwa 70 regional und national agierenden deutschen gesetzlichen Krankenversicherungen (GKV) in der Datenbank des Instituts für angewandte Gesundheitsforschung Berlin (InGef) bereitgestellt wurden. Diese umfassende Datenbank medizinischer und pharmazeutischer Versorgungsleistungen operiert auf anonymisierten longitudinalen Versichertendaten und wurde mit der Statistiksoftware SAS Enterprise Guide, Version 9.2, (SAS Institute Inc., Cary (NC), USA) analysiert. Die Studie umfasste die Baseline-Phase (2011–2012), die Induktionsphase (definiert als das Diagnosequartal im Jahr 2013 plus 2 Quartale nach der Diagnose) sowie 3 Folgejahre (2014–2016). Die nach Alter und Geschlecht stratifizierte Kohorte von 4.040.919 Personen ist repräsentativ für die gesamte deutsche Bevölkerung [1]. Die Analyse der Studienpopulation von etwa 3 Mio. Versicherten war auf die Kohorte der erwachsenen Personen (≥18 Jahre) beschränkt, die zwischen 2011 und 2016 lückenlos beobachtet werden konnten (2.978.136 Personen).

### Patientenauswahlkriterien.

Patienten wurden basierend auf ihrer primären oder sekundären Krankenhausentlassungsdiagnose im Jahr 2013 (Indexdiagnose) als Patienten mit GPA (Internationale statistische Klassifikation der Krankheiten und verwandter Gesundheitsprobleme [ICD-10] M31.3) oder MPA (ICD-10 M31.7) identifiziert, wobei die Diagnose ambulant mindestens in 2 Quartalen eines laufenden Jahres bestätigt werden musste. Patienten mit verwandten Diagnosen – ICD-10 M30, M31 (ohne .3, .7, .9), D69.0, D89.1 oder M35.2 – nach der Indexdiagnose wurden ausgeschlossen. Die prävalente Studienpopulation (einschließlich der Inzidenzfälle) im Indexjahr 2013 umfasste 722 Personen, 601 GPA- und 121 MPA-Patienten. Zusätzlich war bei der inzidenten Studienpopulation 2013 (132 Personen, 95 GPA- und 37 MPA-Patienten) keine Diagnose einer GPA oder MPA in mindestens 8 Quartalen vor der Indexdiagnose erlaubt. Aufgrund der Datenschutzbestimmungen wurden Fälle mit <5 Personen nicht in den Analysen dargestellt. Eine Zusammenstellung aller genannten ICD-10-Codierungen ist in Tab. S1 im Online-Zusatzmaterial zu finden.

### Studienvariablen.

Die 20 am häufigsten auftretenden begleitenden Morbiditäten bei inzidenten GPA- und MPA-Patienten wurden für das Indexjahr auf der Grundlage ihrer ICD-10-Codierung für ambulante oder stationäre primäre und sekundäre Entlassungsdiagnosen ermittelt. Inzidente GPA- und MPA-Patienten mit schwerer Nierenerkrankung wurden definiert durch eine chronische Nierenerkrankung im Stadium III, IV und V (ICD-10 N18.3/.4/.5) sowie durch eine Nierenersatztherapie, codiert als Operationen- und Prozedurenschlüssel (OPS) 8‑853, 8‑854, 8‑855, 8‑856 oder 8‑857, und wurden für die Baseline-Phase, die Induktionsphase und 3 Folgejahre erfasst. Für das Indexjahr wurde zudem der Anteil an prävalenten GPA- und MPA-Patienten mit entsprechender Nierenersatztherapie erhoben. Für die Zählung der Nierentransplantationen (OPS 5‑555) bei prävalenten GPA- und MPA-Patienten wurde der Beobachtungszeitraum des Indexjahrs und der 2 Folgejahre erfasst. Als schwere Infektionen bei inzidenten GPA- und MPA-Patienten wurden Lungenentzündung, Sepsis, Bronchopneumonie, Infektion der unteren Atemwege, Pleuropneumonie und chronisch-obstruktive Lungenerkrankung definiert (ICD-10 J12, J13, J14, J15, J16, J17, J18; J20, J21, J22, J44, R65 als Haupt- oder Sekundärdiagnose für die stationäre Entlassung), die zu einem Krankenhausaufenthalt führten oder sich während des Krankenhausaufenthalts entwickelten. Die Patienten wurden dabei über die Induktionsphase und 3 Folgejahre analysiert. Die Mortalitätsrate pro Quartal wurde für prävalente GPA- und MPA-Patienten gruppiert in (a) mit Nierenbeteiligung ICD-10 N00–N29, (b) mit Infektionen ICD-10 A00–B99, R65, (c) mit pulmonaler Beteiligung ICD-10 J00–J22, J40–J99 und (d) sonstige GPA/MPA-Patienten ohne diese Morbiditäten ermittelt. Die jeweilige Mortalitätsrate wurde über die 3 Folgejahre gemittelt. Die Analyse der ambulanten Verschreibung von Glukokortikoiden sowie der Rate schwerer Rezidive von inzidenten GPA- und MPA-Patienten ist im Online-Zusatzmaterial beschrieben.

## Ergebnisse

### Altersprofil der Granulomatose mit Polyangiitis und mikroskopischer Polyangiitis

Nach der Identifizierung von GPA- und MPA-Patienten aus einer Kohorte von etwa 3 Mio. Personen, die repräsentativ für die deutsche Allgemeinbevölkerung ist (GKV-Durchschnittspopulation), wurde die Altersverteilung dieser Patienten untersucht (Abb. S1 im Online-Zusatzmaterial). Prävalenz und Inzidenz von GPA und MPA nahmen mit dem Alter zu und erreichten in der beobachteten Patientenkohorte bei einem Alter von 70 bis 79 Jahren ein Maximum. Das geschätzte mediane Patientenalter einer inzidenten sowie prävalenten GPA-Erkrankung lag in der Gruppe der 60- bis 69-jährigen Patienten, wobei dies für MPA aufgrund der geringen Patientenzahl nicht ermittelt werden konnte.

### Assoziation von AAV mit vielfältigen Komorbiditäten

**Figure 1:**
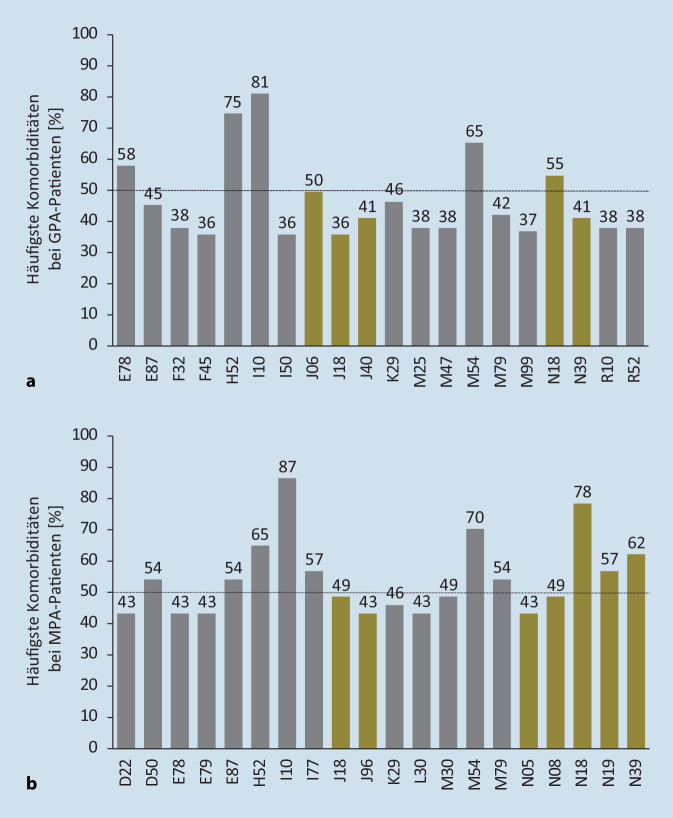

Zur Abschätzung der Krankheitslast von AAV-Patienten wurden anhand der Versorgungsdaten die 20 am häufigsten auftretenden begleitenden Morbiditäten bei inzidenten GPA- und MPA-Patienten anhand der ICD-10-Codierung ermittelt (Abb. 1). Die bei GPA- und MPA-Patienten festgestellten Morbiditäten waren zur Hälfte identisch. Die vorherrschenden begleitenden Morbiditäten waren Hypertonie (I10) bei 81 % bzw. 87 % der GPA- und MPA-Patienten, Nierenerkrankungen (chronische Nierenerkrankung N18 bei 55 % bzw. 78 % der Patienten, Niereninsuffizienz N19, nephritisches Syndrom N05, glomeruläre Störungen N08 oder sonstige Krankheiten des Harnsystems N39) und pulmonale Erkrankungen (Infektion der oberen Atemwege J06, Pneumonie J18, Bronchitis J40 oder respiratorische Insuffizienz J96), die mit bekannten Begleiterscheinungen der AAV übereinstimmen. Des Weiteren traten bei 75 % bzw. 65 % der GPA- und MPA-Patienten Augenerkrankungen (H52) und bei 65 % bzw. 70 % der Patienten Rückenschmerzen (M54) auf, wobei sich unter anderem auch Störungen des Lipoproteinstoffwechsels (E78) und Krankheiten der Arterien und Arteriolen (I77) vermehrt zeigten.

### AAV und schwere Nierenerkrankungen

**Figure 2:**
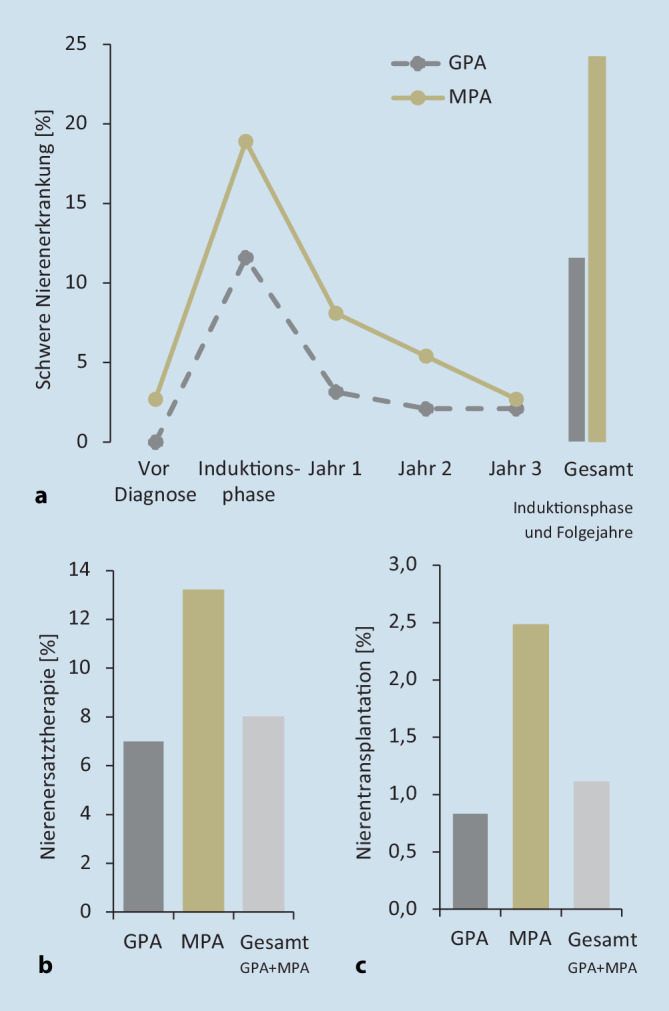

Nach Identifizierung der häufigsten begleitenden Morbiditäten bei AAV-Patienten wurden das Vorkommen und die Auswirkungen einer Nierenschädigung genauer untersucht. Wie aus Abb. 2a ersichtlich, trat eine schwere Nierenerkrankung, definiert als chronische Nierenerkrankung im Stadium III, IV bzw. V oder Nierenersatztherapie, bei 11,6 % der inzidenten GPA- und 24,3 % der inzidenten MPA-Patienten innerhalb von 15 Quartalen nach Diagnose auf. Das Vorkommen war in der Induktionsphase besonders ausgeprägt, wobei die MPA (19 %) eine höhere Wahrscheinlichkeit für schwere Nierenschäden zeigte als die GPA (12 %). In den 3 Folgejahren sanken diese Werte jedoch kontinuierlich auf ein Niveau, das dem vor der Diagnose entsprach. Diese Daten belegen, dass die AAV häufig und vor allem zu Anfang nach der Diagnose mit der Entwicklung einer schweren Nierenbeteiligung assoziiert ist.

Eine Nierenersatztherapie war im Indexjahr bei 7,0 % der GPA- und 13,2 % der MPA-Patienten (insgesamt 8,0 %) notwendig (Abb. 2b). Zudem wurden innerhalb des Beobachtungszeitraums von 3 Jahren nach Diagnose 0,8 % der GPA- und 2,5 % der MPA-Patienten (insgesamt 1,1 %) einer Nierentransplantation unterzogen (Abb. 2c). Dies zeigt erneut, dass MPA- im Vergleich zu GPA-Patienten stärker von einer Nierenbeteiligung betroffen sind.

### AAV und schwere Infektionen

**Figure 3:**
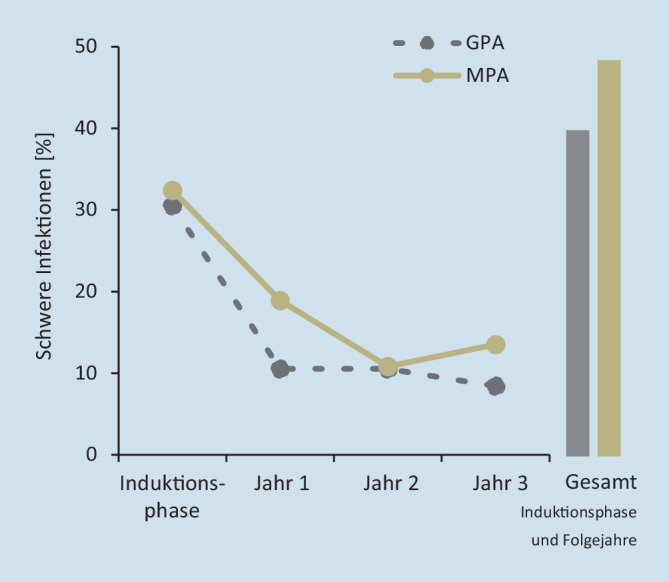

Wie in Abb. 3 gezeigt, entwickelte in der Induktionsphase der AAV-Therapie etwa ein Drittel aller inzidenten Patienten eine schwere Infektion (s. oben, beispielsweise Sepsis oder Atemwegserkrankungen), die eine stationäre Behandlung erforderte oder während des Krankenhausaufenthalts auftrat. In den Folgejahren sank die Häufigkeit schwerer Infektionen mit stationärer Aufnahme auf ca. 10 %. Insgesamt betrachtet hatten GPA- und MPA-Patienten mit 40 % bzw. 49 % ein ähnliches Risiko, innerhalb des gesamten Beobachtungszeitraums eine schwere Infektion zu entwickeln.

### Häufigkeit schwerer AAV-Rezidive

Schwere Rezidive der AAV stellen eine lebensbedrohliche Situation dar und werden wie die akute Erkrankung in der Induktionsphase therapiert. Um die Rate schwerer Rezidive zu ermitteln, wurde die vorübergehende Verschreibung hoch dosierter Glukokortikoide (>60 mg/Tag) in Kombination mit Cyclophosphamid oder Rituximab im Anschluss an eine Phase der Erhaltungstherapie (Gabe niedrig dosierter Glukokortikoide) analysiert (Abb. S2A im Online-Zusatzmaterial). Die Erhebung eines schweren Rezidivs lehnt sich somit an dessen gebräuchliche Definition an, wie sie auch in der S1-Leitlinie zur Diagnostik und Therapie der AAV zu finden ist [18]. Insgesamt hatten 11–12 % der inzidenten Patienten mit GPA und MPA innerhalb von 3 Folgejahren (5–8 % pro Jahr) nach der Induktionsphase ein schweres Rezidiv (Abb. S2B im Online-Zusatzmaterial). Innerhalb der gesamten Beobachtungsphase waren die Raten schwerer Rezidive bei GPA und MPA nahezu vergleichbar.

### Mortalitätsrate von Patienten mit AAV

**Figure 4:**
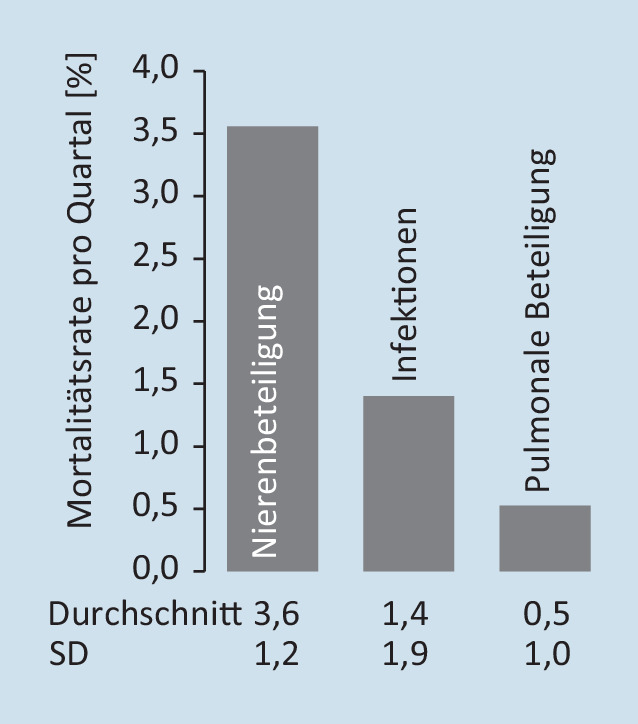

Schließlich wurde die Mortalitätsrate von Patienten mit AAV im Zusammenhang mit begleitenden Morbiditäten untersucht (Abb. 4). Pro Quartal verstarben in der Kohorte durchschnittlich 3,6 % der GPA- und MPA-Patienten, die eine Nierenerkrankung entwickelten (jährliche Mortalität 14,4 %). Somit war eine Nierenbeteiligung der stärkste Faktor in Verbindung mit der Sterblichkeit von Patienten mit AAV. Darüber hinaus wiesen Patienten mit Infektionen sowie einer pulmonalen Beteiligung eine Mortalitätsrate von 1,4 % bzw. 0,5 % pro Quartal auf (jährliche Mortalität 5,6 % bzw. 2,0 %).

## Diskussion

In dieser retrospektiven Versorgungsdatenstudie, die auf einer Kohorte von 3 Mio. Versicherten basiert, wurde die Krankheitslast von Patienten mit AAV bezüglich begleitender Morbiditäten, schwerer Nierenerkrankungen bzw. Infektionen und der Sterblichkeit untersucht. Zuvor konnte gezeigt werden, dass die Population dieser Datenbank in Bezug auf Alter, Geschlecht und regionale Verteilung für die deutsche Gesamtbevölkerung repräsentativ ist [1]. Mögliche Limitationen von Versorgungsdatenstudien bestehen in der eingeschränkten Erfassung klinischer Details, beispielsweise von Laborparametern, wobei die Datenbanken den Vorteil einer immens großen Studienpopulation bieten, welche die Erhebung von Real-world-Evidenz insbesondere im Fall seltener Krankheiten ermöglicht [10]. Die Studienkohorte zeigte eine vergleichbare Altersverteilung von GPA- und MPA-Patienten. Der durchschnittliche Zeitpunkt der GPA-Diagnose stimmt zudem mit früheren Publikationen überein, bei denen sich ein mittleres Alter von etwa 60 Jahren ergab [16, 21].

Die vorliegende Studie bestätigt und unterstreicht Häufigkeit und Auswirkungen einer Nierenbeteiligung

Die 20 am häufigsten auftretenden begleitenden Morbiditäten, die in dieser Studie ermittelt wurden, sind vielfältig. Sie stimmen mit den bekannten Manifestationen von AAV überein (beispielsweise Nierenbeteiligung und pulmonale Erkrankungen). Zudem wurden aber auch begleitende Morbiditäten wie Hypertonie, Augenerkrankungen oder Rückenschmerzen beobachtet, die vermutlich teilweise altersbedingte medizinische Probleme darstellen. Bei den beobachteten begleitenden Morbiditäten handelt es sich daher nicht zwingend um eine direkte Folge der Vaskulitis, da diese Morbiditäten auch infektionsbedingt oder durch den allgemeinen Gesundheitszustand des Patienten bedingt sein können und möglicherweise unabhängig von der AAV aufgetreten sind.

Insbesondere eine Nierenbeteiligung ist mit einer hohen Therapielast verbunden und stellt einen wesentlichen Risikofaktor für einen ungünstigen Verlauf der AAV dar [2, 12]. 19 % der MPA- und 12 % der GPA-Patienten litten innerhalb der Induktionsphase an einer schweren Nierenerkrankung, während bei 13 % der MPA- und 7 % der GPA-Patienten im ersten Untersuchungsjahr eine Nierenersatztherapie erforderlich war. Im Vergleich dazu erhalten in Westeuropa weniger als 0,6 % der Allgemeinbevölkerung ≥75 Jahre eine Nierenersatztherapie [15]. Diese Studie bestätigt und unterstreicht Häufigkeit und Auswirkungen einer Nierenbeteiligung, indem sie auf einen erheblich beeinträchtigten Gesundheitszustand von AAV-Patienten mit der Notwendigkeit regelmäßiger Krankenhausaufenthalte, beispielsweise bedingt durch Nierenersatztherapie oder Nierentransplantation, hinweist.

Schwere Rezidive stellen weiterhin ein Problem im Management von AAV dar

Schwere Infektionen stellen eine weitere Komplikation für Patienten mit AAV dar. In der Induktionsphase entwickelten mehr als 30 % der GPA- und MPA-Patienten während eines Krankenhausaufenthalts schwere Infektionen oder mussten aus diesem Grund stationär behandelt werden. Im Vergleich dazu muss höchstens 1 % der 60- bis unter 65-Jährigen in der Allgemeinbevölkerung aufgrund von Infektionen vollstationär behandelt werden [19]. Die Behandlung mit Glukokortikoiden ist ein zentraler Bestandteil der gegenwärtigen AAV-Therapie, stellt aber aufgrund der immunsuppressiven Wirkung einen hohen Risikofaktor für schwere Infektionen dar [12, 13]. In der Induktionsphase erhielten bis zu 51 % der inzidenten Patienten mit GPA und MPA ambulant mittel oder hoch dosierte Glukokortikoide (Abb. S2 im Online-Zusatzmaterial). Vermutlich trägt eine mittel bis hoch dosierte Glukokortikoidbehandlung wesentlich zu den beobachteten erhöhten Infektionsraten bei, wobei aber auch niedrige Dosierungen die Organfunktionen bei Langzeitbehandlung erheblich beeinträchtigen können [22]. Neben den begleitenden Morbiditäten bedeutet die derzeitige Therapie eine zusätzliche Belastung für Patienten mit AAV, was den dringenden Bedarf an alternativen Therapiestrategien zeigt. Insgesamt betrachtet bestätigt die Auswertung der Versorgungsdaten die bekannten AAV-spezifischen Organbeteiligungen und quantifiziert diese für den deutschen Kontext.

Rezidive bedeuten eine erneute hohe medizinische Belastung für Patienten mit AAV. In früheren Untersuchungen wurden Rezidivraten der AAV von etwa 50 % innerhalb von 5 Jahren festgestellt [17]. Die vorliegende Studie konzentriert sich jedoch auf die Rate schwerer Rezidive, die einen potenziell lebensbedrohlichen Zustand darstellen und eine wiederholte Behandlung mit hoch dosierten Glukokortikoiden (>60 mg/Tag) erfordern [5]. Schwere Rezidive traten jährlich bei 5–8 % der Patienten auf und erhöhen die Krankheitslast somit erheblich. Dagegen werden leichte Rezidive mit niedrigen Glukokortikoiddosierungen behandelt und ereignen sich etwa 3‑mal häufiger als schwere Rezidive [14, 26]. Als Limitation dieser Studie bei Ermittlung der Raten schwerer Rezidive ist jedoch zu sehen, dass deren Abschätzung auf der ambulanten Glukokortikoideinnahme beruht, was zu einer Unterschätzung ihrer Häufigkeit führt, da die individuelle stationäre Verordnung von Glukokortikoiden in den Versorgungsdaten nicht erfasst wird. Die vorliegende Studie zeigt, dass schwere Rezidive weiterhin ein Problem im Management von AAV darstellen. Eine quantitative Erhebung der Häufigkeit schwerer Rezidive sollte jedoch unter Einbeziehung der stationären Glukokortikoidverschreibung erfolgen.

Die Mortalitätsrate von Patienten mit AAV wurde in Abhängigkeit vom Auftreten begleitender Morbiditäten analysiert. Die höchste Sterblichkeitsrate von jährlich 14,4 % wurde bei Patienten mit Nierenbeteiligung festgestellt, gefolgt von Patienten, die an Infektionen litten. Diese Zahlen stehen im Einklang mit einer Gesamtmortalität von etwa 13 %, wie sie bereits früher für Patienten mit AAV berichtet wurde [3]. Im Vergleich sterben in der deutschen Allgemeinbevölkerung jährlich nur 0,9 % der 60- bis unter 65-jährigen Personen (Durchschnittsalter der Patienten mit AAV), sodass bei diesen Patienten mit AAV eine deutlich erhöhte Sterblichkeit im Vergleich zu gleichaltrigen Personen vorliegt [20].

Die Rezidiv- und Sterblichkeitsraten von Patienten mit AAV sind immer noch alarmierend hoch

Angesichts der derzeitigen Therapiemöglichkeiten sind die Rezidiv- und Sterblichkeitsraten von Patienten mit AAV immer noch alarmierend hoch. Daher werden zur Behandlung der AAV innovative Therapieansätze benötigt. In den letzten Jahren wurden verschiedene aussichtsreiche Studien zur Senkung der Glukokortikoiddosierung [22] und mit dem C5a-Rezeptor-Inhibitor Avacopan durchgeführt [8], mit dessen klinischer Anwendung die Belastung durch AAV zukünftig möglicherweise verringert werden kann.

## Fazit für die Praxis

Diese Studie trägt zu einem detaillierten Verständnis der hohen Krankheitslast von Patienten mit AAV bei. Anhand der Analyse deutscher Versorgungsdaten wurden, basierend auf einer Kohorte von etwa 3 Mio. Versicherten, krankheitsspezifische Annahmen der Belastung von Patienten mit AAV bestätigt und für den deutschen Kontext spezifiziert. Patienten mit AAV sind dem Risiko ernsthafter Komplikationen ausgesetzt, die sich aus diversen begleitenden Morbiditäten (insbesondere Nierenschädigungen) sowie aus schweren Infektionen im Zusammenhang mit den Nebenwirkungen der AAV-Therapie (Einnahme von Glukokortikoiden) ergeben und das Leben der Patienten erheblich beeinträchtigen. Daher sind innovative Therapieansätze zur Verringerung der Rezidiv- und Mortalitätsraten erforderlich, um die Belastung durch AAV zu verringern.

## Supplementary Information






