# Supplementary material for: High burden of disease in patients with ANCA-associated vasculitis: A claims data study in Germany
Source: Internist (Berl). 2021 Oct 19;63(2):210–6. [Article in German] doi: 10.1007/s00108-021-01181-z (PMC8813869; doi:10.1007/s00108-021-01181-z)
Supplement: Supplementary file 1 [file 108_2021_1181_MOESM1_ESM.pdf]

Online-Zusatzmaterial

Tab. S1: Übersicht über die verwendeten ICD-10 Kodierungen mit entsprechender Erkrankung bzw. Erkrankungsgruppe.

ICD-10	Erkrankung
A00-B99	Bestimmte infektiöse und parasitäre Krankheiten
D22	Melanozytennävus
D50	Eisenmangelanämie
D69.0	Purpura anaphylactoides<br>inkl. allergische Vaskulitis, Purpura: allergica, Purpura: nicht-thrombozytopenisch: hämorrhagisch, Purpura: nicht-thrombozytopenisch: idiopathisch, Purpura: Schoenlein-Henoch, Purpura: vaskulär
D89.1	Kryoglobulinämie<br>inkl. Kryoglobulinämie: essentiell, Kryoglobulinämie: gemischt, Kryoglobulinämie: idiopathisch, Kryoglobulinämie: primär, Kryoglobulinämie: sekundär, Kryoglobulinämische: Purpura, Kryoglobulinämische: Vaskulitis
E78	Störungen des Lipoproteinstoffwechsels und sonstige Lipidämien
E79	Störungen des Purin- und Pyrimidinstoffwechsels
E87	Sonstige Störungen des Wasser- und Elektrolythaushaltes sowie des Säure-Basen-Gleichgewichts
F32	Depressive Episode
F45	Somatoforme Störungen
H52	Akkommodationsstörungen und Refraktionsfehler
I10	Essentielle (primäre) Hypertonie
I50	Herzinsuffizienz
I77	Sonstige Krankheiten der Arterien und Arteriolen
J00-J22, J40-J99	Akute Infektionen der oberen Atemwege, Grippe und Pneumonie, sonstige akute Infektionen der unteren Atemwege, chronische Krankheiten der unteren Atemwege, Lungenkrankheiten durch exogene Substanzen, sonstige Krankheiten der Atmungsorgane, die hauptsächlich das Interstitium betreffen, Purulente und nekrotisierende Krankheitszustände der unteren Atemwege, sonstige Krankheiten der Pleura, sonstige Krankheiten des Atmungssystems
J06	Akute Infektionen an mehreren oder nicht näher bezeichneten Lokalisationen der oberen Atemwege
J12-J18; J20-J22, J44	Viruspneumonie, anderenorts nicht klassifiziert, Pneumonie durch Streptococcus pneumoniae, Pneumonie durch Haemophilus influenzae, Pneumonie durch Bakterien, anderenorts nicht klassifiziert, Pneumonie durch sonstige Infektionserreger, anderenorts nicht klassifiziert, Pneumonie bei anderenorts klassifizierten Krankheiten, Pneumonie, Erreger nicht näher bezeichnet, akute Bronchitis, akute Bronchiolitis, akute Infektion der unteren Atemwege, nicht näher bezeichnet, sonstige chronische obstruktive Lungenkrankheit
J18	Pneumonie, Erreger nicht näher bezeichnet
J40	Bronchitis, nicht als akut oder chronisch bezeichnet
J96	Respiratorische Insuffizienz, anderenorts nicht klassifiziert
K29	Gastritis und Duodenitis
L30	Sonstige Dermatitis
M25	Sonstige Gelenkkrankheiten, anderenorts nicht klassifiziert
M30	Panarteriitis nodosa und verwandte Zustände
M31	Sonstige nekrotisierende Vaskulopathien

M31.3	Granulomatose mit Polyangiitis (GPA)/Wegener-Granulomatose<br>inkl. Granulomatose mit Polyangiitis mit Lungenbeteiligung (J99.1), Granulomatose mit Polyangiitis mit Nierenbeteiligung (N08.5), nekrotisierende Granulomatose der Atemwege
M31.7	Mikroskopische Polyangiitis (MPA)<br>inkl. mikroskopische Polyarteriitis, exkl. Polyarteriitis nodosa (M30.0)
M31.9	Nekrotisierende Vaskulopathie, nicht näher bezeichnet
M35.2	Behçet-Krankheit
M47	Spondylose
M54	Rückenschmerzen
M79	Sonstige Krankheiten des Weichteilgewebes, anderenorts nicht klassifiziert
M99	Biomechanische Funktionsstörungen, anderenorts nicht klassifiziert
N00-N29	Glomeruläre Krankheiten, tubulointerstitielle Nierenkrankheiten, Niereninsuffizienz, Urolithiasis, sonstige Krankheiten der Niere und des Ureters
N05	Nicht näher bezeichnetes nephritisches Syndrom
N08	Glomeruläre Krankheiten bei anderenorts klassifizierten Krankheiten
N18	Chronische Nierenkrankheit
N18.3/.4/.5	Chronische Nierenkrankheit Stadium 3<br>inkl. glomeruläre Filtrationsrate (GFR) 30 bis unter 60 ml/min/1,73 m^2 Körperoberfläche<br>Chronische Nierenkrankheit Stadium 4<br>inkl. GFR 15 bis unter 30 ml/min/1,73 m^2 Körperoberfläche (präterminale Niereninsuffizienz)<br>Chronische Nierenkrankheit Stadium 5<br>inkl. dialysepflichtige chronische Niereninsuffizienz, GFR unter 15 ml/min/1,73 m^2 Körperoberfläche (terminale Niereninsuffizienz)
N19	Nicht näher bezeichnete Niereninsuffizienz
N39	Sonstige Krankheiten des Harnsystems
R10	Bauch- und Beckenschmerzen
R52	Schmerz, anderenorts nicht klassifiziert
R65	Systemisches inflammatorisches Response-Syndrom

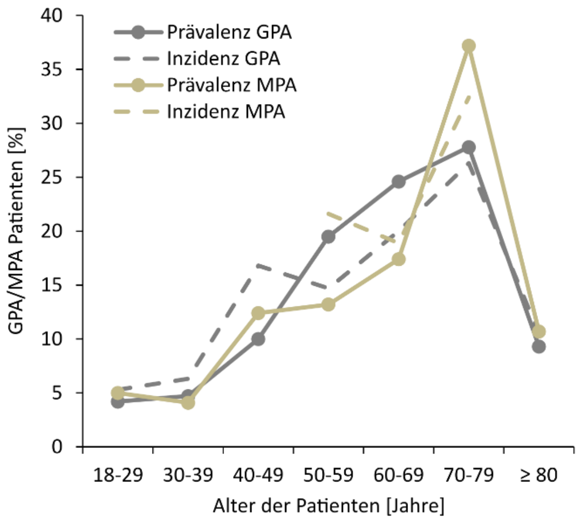

Abb. S1: Altersverteilung der prävalenten und inzidenten GPA- und MPA-Patienten.
Für die Bestimmung der Altersverteilung bei prävalenten und inzidenten GPA- und MPA-Patienten wurde die entsprechende Studienpopulation für das Indexjahr ausgewertet. Unterhalb von 50 Jahren und oberhalb von 80 Jahren lag das Inzidenzrisiko für MPA unter der Empfindlichkeitsschwelle von < 5 Personen.

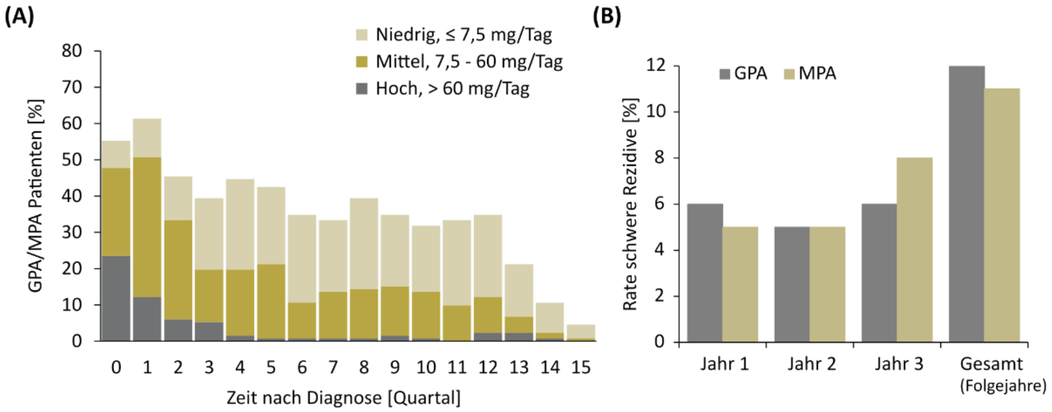

Abb. S2: (A) Ambulant verordnete Glukokortikoide bei inzidenten GPA- und MPA-Patienten ab Diagnose bis 15 Quartale nach Diagnose.

Für inzidente GPA- und MPA-Patienten wurde die Dosierung der Glukokortikoide pro Quartal berechnet, beginnend mit dem Quartal der Diagnose unter Verwendung der PZN-Code-Information für die Substanz H02AB (Glukokortikoide). Jede Dosisberechnung erstreckte sich über die erste und die letzte Verschreibung (mindestens 2) innerhalb des entsprechenden Beobachtungsintervalls. Die Anzahl der Tage zwischen der ersten und der letzten Verschreibung wurde zur Berechnung der Tagesdosis herangezogen, wobei die letzte Verschreibung des Intervalls von der Berechnung ausgeschlossen wurde. Die Menge wurde dann in 4 Eskalationsstufen eingeteilt (keine Verschreibung; ≤ 7,5 mg/Tag „niedrig“; 7,5 - 60 mg/Tag „mittel“; > 60 mg/Tag „hoch“ in Bezug auf Prednison-Äquivalente). Im Falle einer Änderung der Verschreibung innerhalb eines Quartals wurde nur die höchste Eskalationsstufe berücksichtigt. Die stationäre Verschreibung von Glukokortikoiden wurde in dieser Studie nicht untersucht.

(B) Rate schwerer Rezidive bei inzidenten GPA- und MPA-Patienten über 3 Folgejahre.

Schwere Rezidive von inzidenten GPA- und MPA-Patienten wurden im Anschluss an die Induktionsphase für jedes Folgejahr ermittelt und als Verschreibung von hoch dosierten Glukokortikoiden (> 60 mg/Tag Prednison-Äquivalente) in Kombination mit Cyclophosphamid oder Rituximab nach einer Periode niedrig dosierter Glukokortikoide (Erhaltungstherapie) definiert. Die Erhebung eines schweren Rezidivs lehnt sich somit an dessen gebräuchliche Definition an, wie sie auch in der S1-Leitlinie zur Diagnostik und Therapie der AAV zu finden ist (Schirmer et al., Z Rheumatol 2017).
