# Supplementary material for: High burden of disease in patients with ANCA-associated vasculitis: A claims data study in Germany
Source: Internist (Berl). 2021 Oct 19;63(2):210–6. [Article in German] doi: 10.1007/s00108-021-01181-z (PMC8813869; doi:10.1007/s00108-021-01181-z)
Supplement: Supplementary file 2 [file 108_2021_1181_MOESM2_ESM.pptx]

## Slide 1
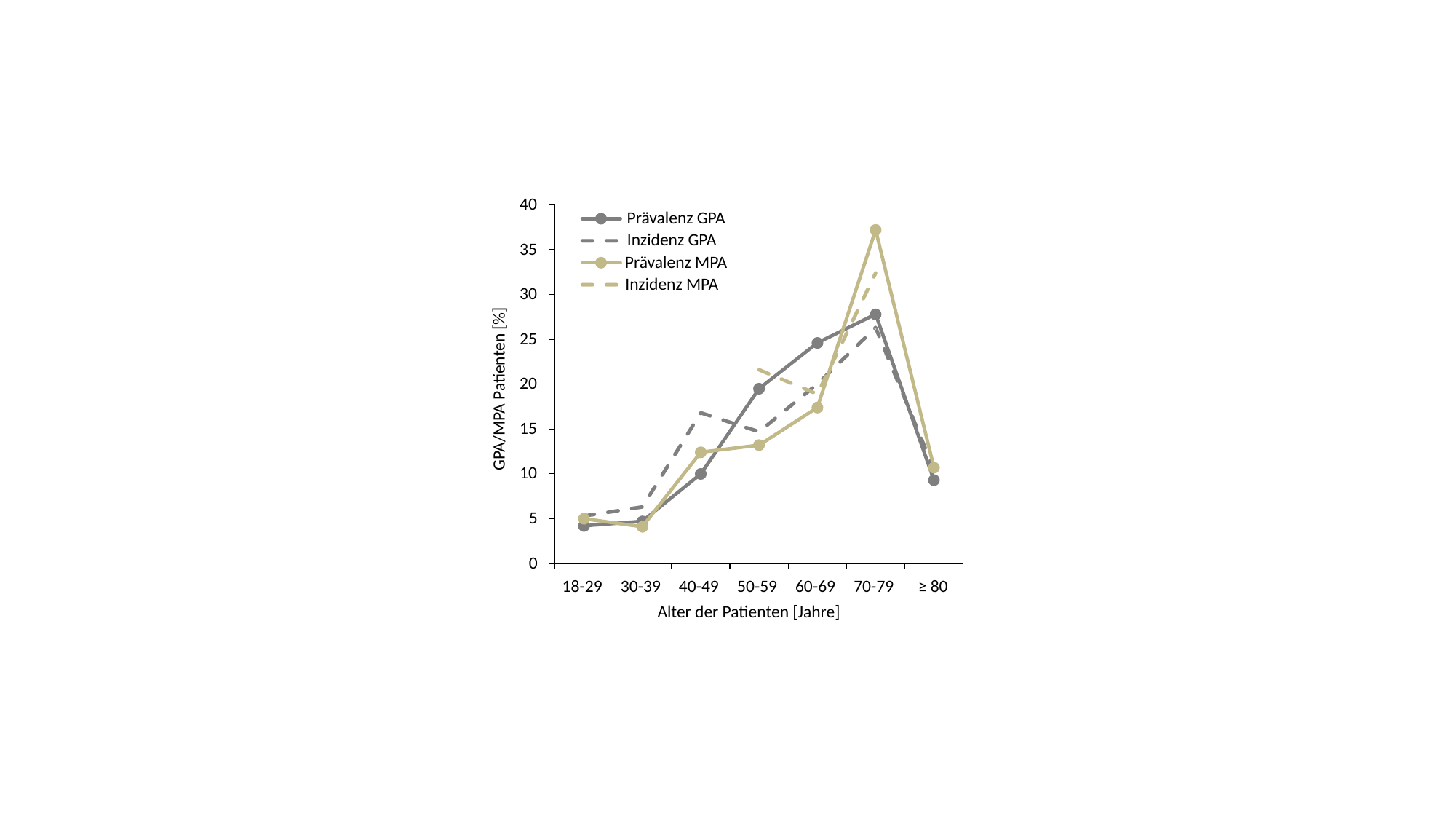

40
Prävalenz GPA
Inzidenz GPA
35
Prävalenz MPA
Inzidenz MPA
30
25
20
GPA/MPA Patienten [%]
15
10
5
0
18-29
30-39
40-49
50-59
60-69
70-79
≥ 80
Alter der Patienten [Jahre]
