# Supplementary material for: High burden of disease in patients with ANCA-associated vasculitis: A claims data study in Germany
Source: Internist (Berl). 2021 Oct 19;63(2):210–6. [Article in German] doi: 10.1007/s00108-021-01181-z (PMC8813869; doi:10.1007/s00108-021-01181-z)
Supplement: Supplementary file 3 [file 108_2021_1181_MOESM3_ESM.pptx]

## Slide 1
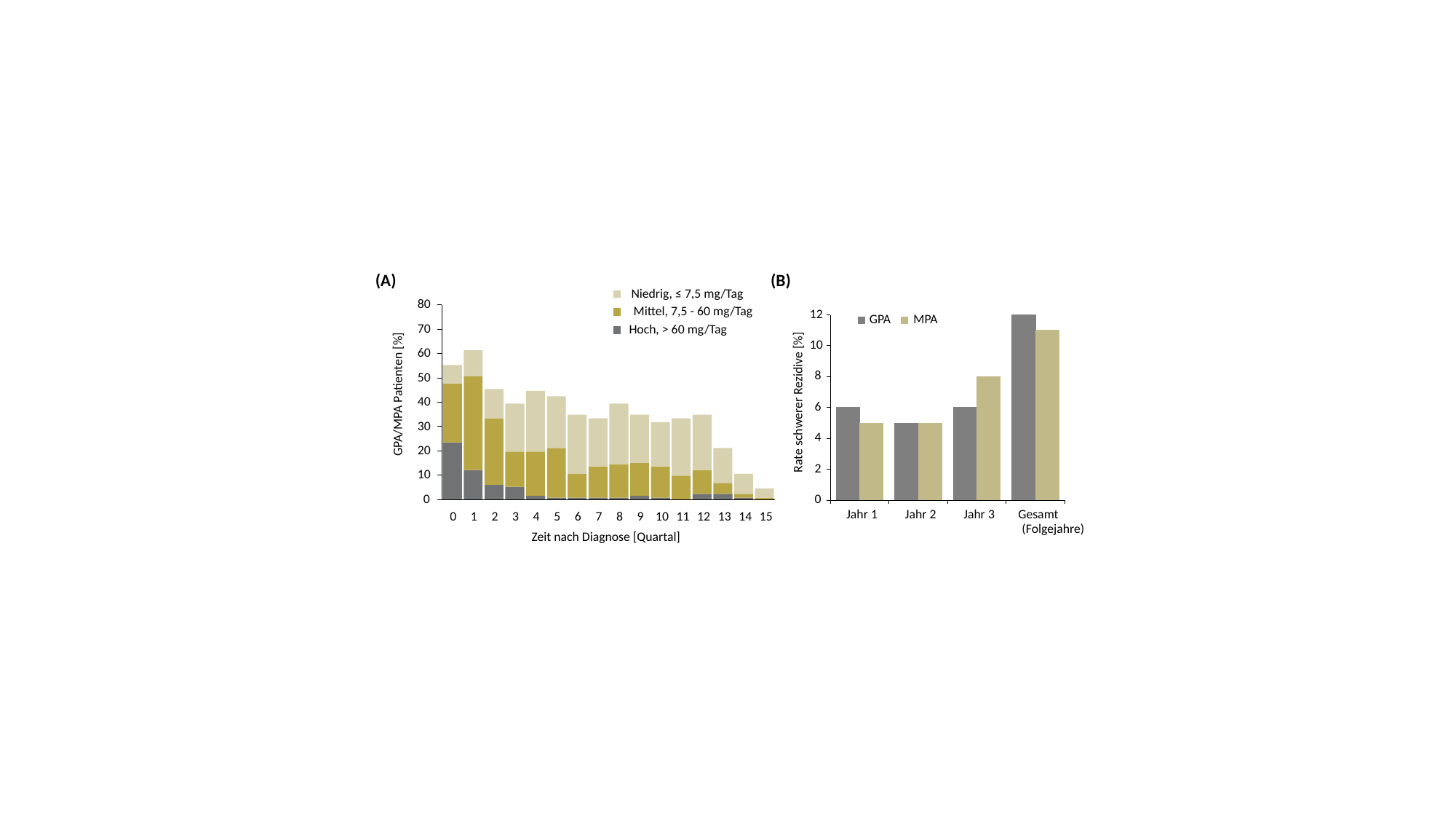

(A)
(B)
Niedrig, ≤ 7,5 mg/Tag
80
Mittel, 7,5 - 60 mg/Tag
12
GPA
MPA
70
Hoch, > 60 mg/Tag
10
60
8
50
GPA/MPA Patienten [%]
40
Rate schwerer Rezidive [%]
6
30
4
20
2
10
0
0
Jahr 1
Jahr 2
Jahr 3
Gesamt
0
1
2
3
4
5
6
7
8
9
10
11
12
13
14
15
(Folgejahre)
Zeit nach Diagnose [Quartal]
